# Impact of Heat Treatment on the Structural, Optical, Magnetic and Photocatalytic Properties of Nickel Oxide Nanoparticles

**DOI:** 10.3390/ma17164146

**Published:** 2024-08-22

**Authors:** Gharam A. Alharshan, A. Almohammedi, M. A. M. Uosif, E. R. Shaaban, M. Emam-Ismail

**Affiliations:** 1 Physics Department, College of Science, Princess Nourah bint Abdulrahman University, P.O. Box 84428, Riyadh 11671, Saudi Arabia; 2 Physics Department, Faculty of Science, Islamic University of Madinah, Almadinah Al-Munawarah 42351, Saudi Arabiaesam_ramadan2008@yahoo.com (E.R.S.); 3 Physics Department, College of Science, Jouf University, Sakaka P.O. Box 2014, Saudi Arabia; 4 Physics Department, Faculty of Science, Al-Azhar University, Assiut 71542, Egypt; 5 Spectroscopy of Polarized Light Laboratory, Physics Department, Faculty of Science, Galala University, New Galala City 43511, Egypt; 6 Thin Films Laboratory, Physics Department, Faculty of Science, Ain Shams University, Cairo 11566, Egypt

**Keywords:** nickel oxide nanoparticles, thermogravimetric analysis, structural analysis, optical properties, photocatalyst

## Abstract

The precursor nanoparticles of nickel hydroxide (Ni(OH)_2_) and nickel oxide (NiO) were successfully converted into the latter by the reaction of nickel chloride with hydrazine at ambient temperature. (TGA) and (DSC) were adapted for annealing the precursor products at different annealing temperatures (210, 285, 350, 390, 425, and 450 °C). XRD, TEM, and UV-VIS absorption spectroscopy were used to characterize the products. Both the band edge and energy gap values decrease with increasing annealing temperatures. Hysteresis loops are visible in the M-H curves of annealed (350 °C and 390 °C) precursor NiO NPs, indicating the presence of ferromagnetic Ni domains. However, NiO nanoparticles annealed at higher temperatures (425 °C and 450 °C) had a straight M-H curve, indicating paramagnetic properties. NiO NPs were used to study photocatalysis in the degradation of the MB dye. As annealing temperatures increased, the catalyst caused the degradation of MB. The sample that was annealed at 450 °C, however, exhibits the maximum photocatalytic activity, reaching up to 72.4% after being exposed to visible light. In other words, it was discovered that as the catalyst’s annealing temperature rose, so did the rate of MB’s photocatalytic degradation.

## 1. Introduction

In recent years, the field of nanotechnology has witnessed significant advancements, particularly in the synthesis and characterization of nanoparticles with tailored properties for various applications [1]. Among these, nickel oxide nanoparticles (NiO NPs) have garnered substantial attention due to their unique structural, optical, magnetic, and photocatalytic properties [2]. These properties can be finely tuned through heat treatment, a critical process influencing the structural evolution and functional characteristics of NiO NPs [3].

Nickel oxide, a p-type semiconductor, exhibits a range of fascinating properties that make it suitable for diverse applications such as gas sensors, catalysts, energy storage devices, and optoelectronic devices [4,5,6]. The ability to manipulate these properties through controlled heat treatment offers researchers the opportunity to enhance NiO NP performance in specific applications [7,8]. Heat treatment not only affects the crystallinity and phase composition but also influences the morphology, size distribution, and surface chemistry of the nanoparticles, thereby impacting their optical absorption, magnetic susceptibility, and photocatalytic activity [9].

In order to create nanosized nickel oxide, a variety of techniques have been tried, including nanoparticles [10], nanorings [11], nanosheets [12], and nanoribbons [12]. For the removal of color from wastewater, there are numerous physical, chemical, and biological approaches [13,14,15,16,17]. An alternate method of purifying water is the advanced oxidation process. Since the photocatalytic process uses oxidizing species such as hydroxyl radicals to break down the organic effluent, it appears to be an effective instrument when light is present. Because of its simplicity and efficiency, adsorption is another approach that holds a lot of promise for the removal of pollutants. As adsorbents, carbon-based materials and other metal oxides were employed. The leaching of the redox metal ions from the adsorbent causes intermediate metal complexes, which are unable to adsorb organic contaminants, resulting in decreased dye removal efficiency even if they are highly reactive with the organic effluent [18,19].

Studies have shown that varying the annealing temperature, duration, and atmosphere during heat treatment can lead to significant changes in the structural integrity of NiO NPs, affecting their bandgap energy, magnetic ordering, and catalytic efficiency. For instance, high-temperature annealing tends to promote crystallization and phase transformation, influencing the optical bandgap and magnetic properties of the nanoparticles. Moreover, the surface defects and oxygen vacancies induced by heat treatment play a crucial role in modulating the photocatalytic behavior of NiO NPs, making them promising candidates for environmental remediation and solar energy conversion applications [20,21]. NiO NPs are much more effective as a catalyst or adsorbent, according to El-Kemary et al. [22], because of their superior durability, strong photosensitivity, and high adsorptive affinity.

The primary aim of this research is to develop strategies for controlling the catalytic activity of nickel oxide (NiO) nanoparticles (NPs) and to identify effective, environmentally responsive materials and technologies for catalytic applications. Heat treatment plays a crucial role in altering the structural, optical, and magnetic properties of NiO NPs, including their crystallite size. Adjusting the band gaps, typically in the nanometer range, is essential for optimizing catalysts’ performance in dye removal and degradation. Thermal processing has been employed to modify the optical and structural characteristics of metal oxide NPs, thereby enhancing their adsorption and catalytic effectiveness. In investigations of the photocatalytic degradation of pollutants, such as methylene blue (MB) dye, variations in the annealing temperature of the NiO precursor have demonstrated significant impacts on the nanoparticles’ structural, morphological, optical, magnetic, and photocatalytic properties.

## 2. Experimental

Ni(OH)_2_ underwent heat breakdown to produce NiO nanoparticles. Precursors included adding hydrazine monohydrate solution (6.73 mL of molar ratio 5) to nickel chloride hexahydrate solution (0.111 M) in absolute ethanol. A pH adjustment of 9 was made using potassium hydroxide. At room temperature, the reaction was agitated for two hours. The finished product was extensively cleaned in acetone and deionized water to remove any reaction remnants. Finally, [Ni(OH)_2_ 0.5H_2_O] nanoparticles were produced and vacuum-dried. The precursor products were annealed at various annealing temperatures (210, 285, 350, 390, 425, and 450 °C) in terms of differential scanning calorimetry (DSC) and thermogravimetric analysis (TGA) (Shimadzu 50 with an accuracy of 0.1 K). To obtain superfine NiO particles, the calcination temperature was set at 450 °C. Using an X-ray powder diffraction (XRD) Shimadzu Diffractometer XRD 6000, (Tokyo, Japan) using Cu-K1 radiation (λ = 1.54056), the structure and phases of the powder sample were determined. Si (99.9999%) pure silicon was employed as the internal standard. Using a JEOL 2010 microscope (Tokyo, Japan) set to an accelerating voltage of 200 kV, transmission electron micrographs (TEM) were produced. Based on spectral absorbance measured using a JASCO V-670 double-beam spectrophotometer (Tokyo, Japan), optical characterization of the powder sample has been carried out. The measurements were made between the wavelengths of 300 and 800 nm.

### Catalyst Characterization

The catalytic test was conducted using a solution of MB at a dosage of 10 mg per liter of water. A total of 50 mL of MB solution and 25 mg of composite were combined using a magnetic stirrer. With constant stirring, one portion of the suspension was kept in the dark, while the other portion was exposed to white light (from a 500 W Xe lamp) at room temperature at regular intervals, followed by UV spectrometer analysis and centrifugation to separate the net analyses.

## 3. Results and Discussion

### 3.1. TGA and DTA Analysis

**Figure 1:**
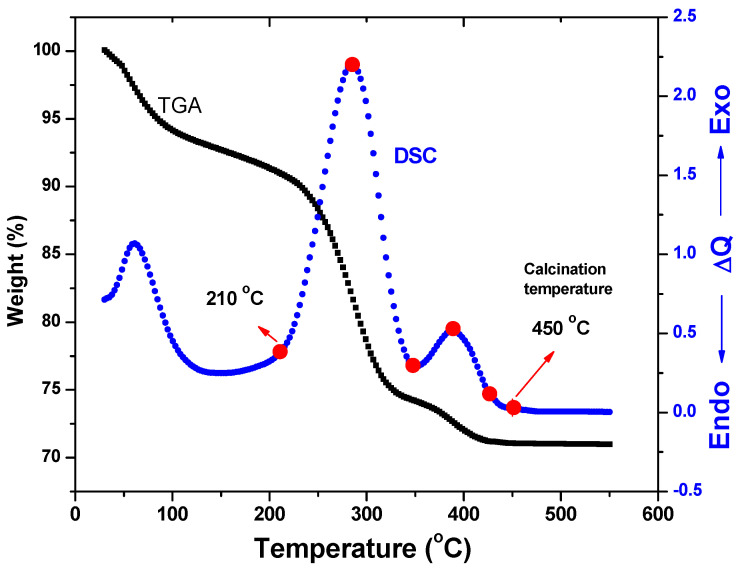
TGA and DSC against the temperature of NiO NPs.

TGA analysis was performed from 25 to 550 °C in the atmosphere to demonstrate the changes that occurred during heat treatment of the precursor powders (Figure 1). It is clear from the TGA curve that there was more than one stage of weight loss. It is possible to attribute the first weight loss (reported to be 6.65 wt%) in the temperature range of 30–210 °C to the evaporation of half the water molecule used in crystallization. Between 215 °C and 345 °C, the precursor loses around 15% of its weight, which is due to the precursor’s thermal disintegration into NiO nanoparticles, which is followed by a significant exothermic DSC peak. Between 350 °C and 435 °C, the precursor loses weight by around 3.5 wt%. This weight loss is followed by a modest exothermic DSC peak that is linked to the formation of flaws in the NiO powder. These flaws are linked to oxygen loss, which promotes the growth of oxygen vacancies. Evidently, the weight loss becomes relatively minimal at 450 °C, indicating that breakdown is complete and NiO nanoparticle production is almost complete.

### 3.2. XRD Analysis

**Figure 2:**
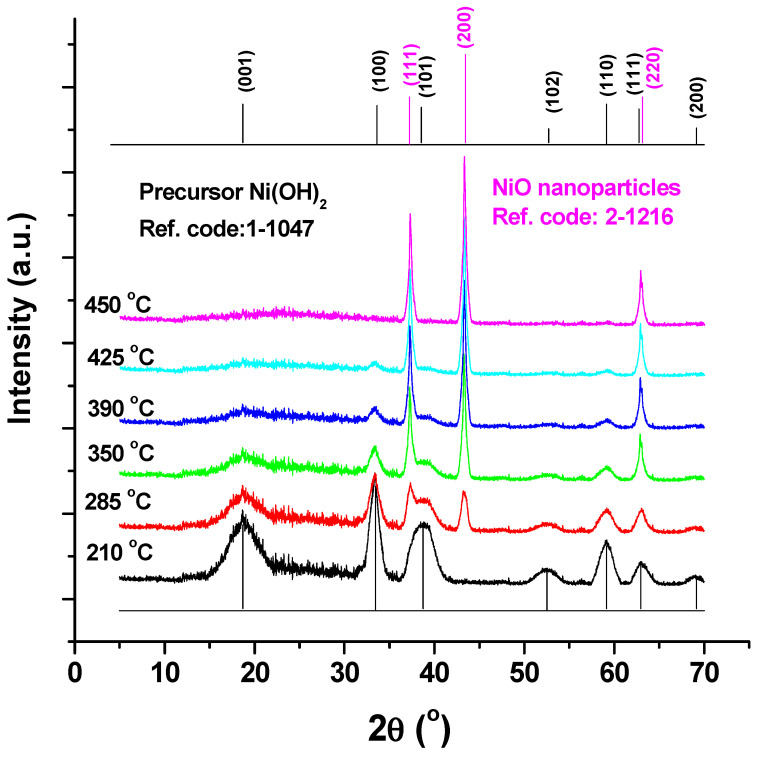
XRD pattern of NiO NPs at varies annealing temperatures.

The precursor Ni(OH)_2_ and NiO nanoparticle products’ XRD patterns are displayed in Figure 2 at various annealing temperatures (210, 285, 350, 390, 425, and 450 °C). The acquired peaks for the hydroxide precursor appear to be noticeably broad, which suggests that the hydroxide crystallites may be in the nanoscale range. Peak broadening variations are thought to be influenced by crystallite form, flaws, and differences in crystal symmetry, in addition to crystallinity. The nuclei expand into larger nanocrystals as the annealing temperature is raised, increasing the crystalline volume and indicating high crystallinity. Additionally, it is clear that as the temperature is increased, the XRD peak broadening (FWHM) narrows and the diffraction peaks intensify, indicating an increase in NiO NP size. The annealed sample’s XRD patterns showed sharpened reflection peaks, which show that NiO’s crystallite sizes have grown. The peak locations appearing at 2θ° = 18.99, 33.15, 38.61, 52.21, 59.18, 62.73 and 69.58 can be indexed as (001), (100), (101), (102), (110), and (111) planes of precursor Ni(OH)_2_ (at 210 °C) according to JCPDS card (no. 1-1047). All reflections of the precursor Ni(OH)_2_ can be indexed as hexagonal with lattice constants (a = b = 3.07 Å and c = 4.6 Å).

Additionally, the peaks observed at 2θ° = 37.09, 43.31, and 62.79 are easily indexed as the (111), (200), and (220) bulk NiO crystal planes. The standard data (JCPDS card no. 47-1049) and all of the reflections may be correlated to the face-centered cubic (fcc) NiO phase with a lattice constant (a): 4.175 [22]. The peaks’ clarity and intensity show that the produced sample is well-crystalline. Ni(OH)_2_ was entirely degraded to NiO at 450 °C, according to the XRD results, which are also supported by the TGA and DSC measurements.

The crystal size (*D*) is inversely proportional to the width of the peak, i.e., decreases according to the following Scherrer equation [23,24,25]

(1) $$D=\frac{0.9\lambda }{\beta \cos\theta }$$

where the integral X-ray peak profile width is provided as *β*, where *β* is the width of the peak equivalent to the difference between the sample and the standard silicon in order to remove the instrumental broadening from the sample peaks.

(2) $$\beta =\sqrt{\beta_{obs}^{2}-\beta_{std}^{2}}.$$

**Figure 3:**
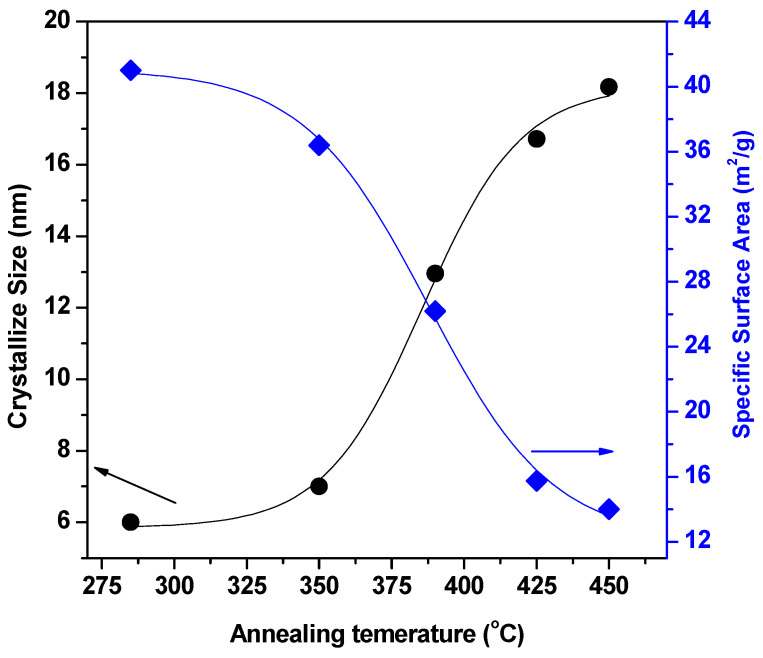
Effect of annealing temperature on crystallite size and specific surface area (SSA) of NiO NPs.

Figure 3 displays the virtual look of the crystallite size (*D*) of the precursor products at different annealing temperatures (210, 285, 350, 390, 425 and 450 °C). According to Figure 3, the treated materials’ predicted crystallization sizes ranged from 6 to 18 nm. With an increase in annealing temperature, crystallite size grows. The nuclei expand into larger nanocrystals as the temperature rises, increasing the crystalline volume and indicating high crystallinity. Additionally, it is clear that as temperature is increased, the XRD peak broadening (FWHM) narrows and the diffraction peaks intensify, indicating an increase in NiO NP size.

The specific surface area (SSA) is the total surface area of a solid material per unit of mass. The specific surface area (SSA) can be calculated in terms of the XRD pattern of the samples via crystallite size from the following equation:

(3) $$SSA=\frac{6000}{\rho .D}$$

where ρ is the density in g/cm^3^ and *D* is the average size of the precursor nanoparticle. Figure 3 shows the effect of calcined temperature on the specific surface area (SSA) of NiO NPs. *SSA* is inversely proportional to the crystallite size of the photocatalyst. Figure 3 shows the calculated data for the crystallite sizes and SSA for each annealing temperature of the precursor nanoparticle. The photocatalytic activity of the photocatalyst is dependent on the crystallite size and surface area [26,27,28]. The small crystallite size increases the surface area of the photocatalyst, resulting in increases in the photocatalyst’s adsorption efficiency to the reactant and absorption to the light source [29,30,31].

### 3.3. TEM Analysis

**Figure 4:**
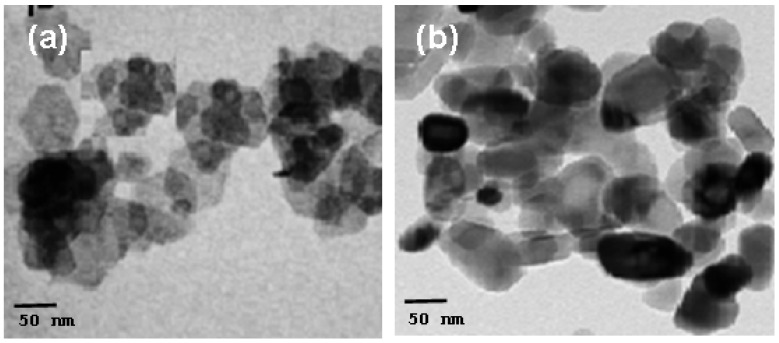
TEM image of (**a**) precursor and (**b**) annealed precursor at (450 °C).

TEM was used to determine the nanoparticles’ size and shape. Figure 4a,b displays typical TEM images of Ni(OH)_2_ and NiO nanoparticles. The precursor Ni(OH)_2_ can be seen in the TEM image as homogeneous particles with a hexagonal shape and a size of about 7 nm. TEM images of NiO nanoparticles, however, show a non-spherical particle shape with smooth and homogeneous particle morphology, with a size (considered average size) that is approximately 18 nm (Figure 4b). We note that the average crystallite size computed using Scherrer’s equation from the XRD pattern and the mean particle size determined by TEM measurements are reasonably in accord.

### 3.4. Optical Characterization

**Figure 5:**
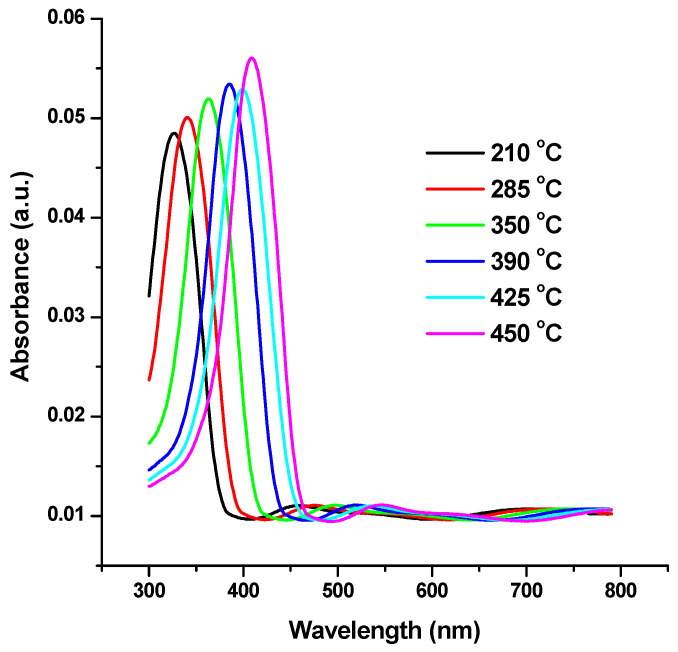
UV-Vis spectra of NiO NPs.

Measurements of optical absorption are crucial for analyzing semiconductor nanostructure dynamics. One theory holds that the fundamental characteristic of semiconductors is their energy band gap. Figure 5 displays the absorbance spectra of the precursor nanoparticles at various annealing temperatures (210, 285, 350, 390, 425, and 450 °C). With rising annealing temperatures, it is shown that the absorption maximum, λ_max_, exhibits red shifts to longer wavelengths, from 327 nm to 409 nm [32,33,34].

**Figure 6:**
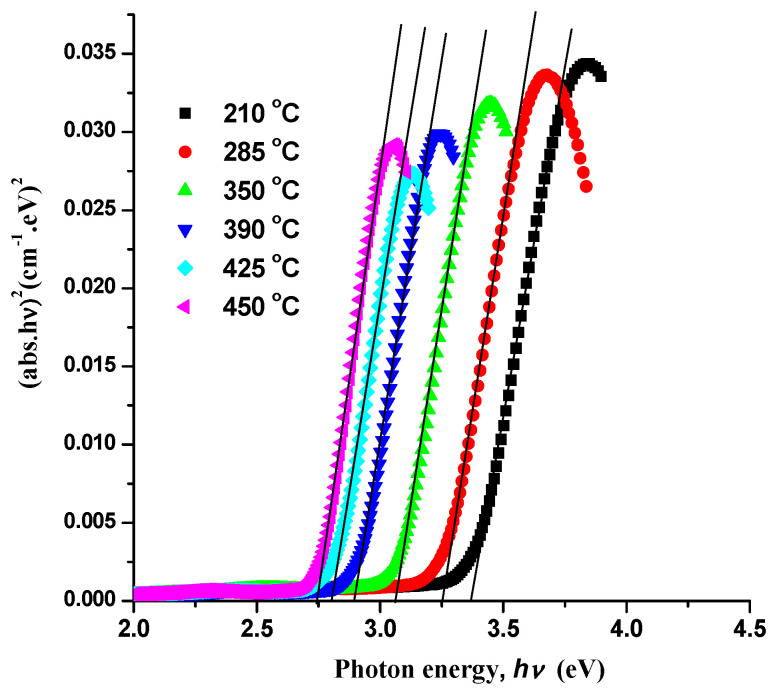
Dependence of (abs. *h*ν)^2^ on the photon energy(*h*ν) for the investigated samples.

**Figure 7:**
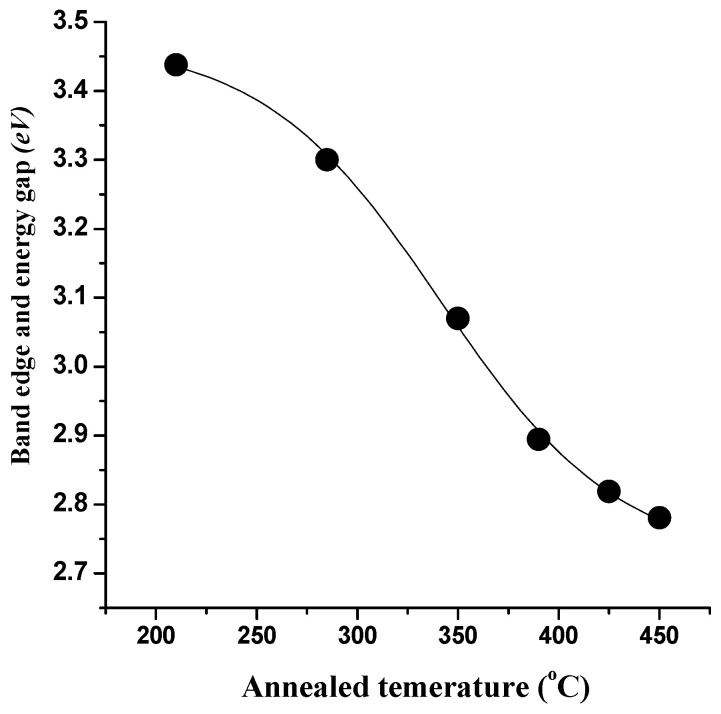
Energy gap as a function of annealed temperature of NiO NPs.

According to Tauc’s relation αhυ = Á (*hυ* − $E_{g}^{opt}$)^m^, where *h* is the Plank constant, υ is the frequency of incident radiation, α is the coefficient of absorption (which is equal to absorbance because the thickness of the cell is 1 cm), and m is considered to be ½ for direct band gap semiconductors, it is possible to estimate band gap and determine the optical properties of prepared precursor nanoparticles through optical absorption measurements. The values of band gaps for manufactured nanoparticles are obtained by extrapolating the linear section of the curve between hυ and (αhυ)^2^ (see Figure 6). For precursor nanoparticles annealed at 210 °C, the predicted band gap value is 3.43 eV, and it drops to 2.78 eV at 450 °C. Figure 7 shows the band edge and energy gap values in relation to the annealing temperatures. Due to the larger crystallites and narrower energy gap, the optical band gap value decreases as the annealing temperature rises.

As the annealing temperature, *T*, rises, the interatomic spacing of the crystallite structure is reduced. 

### 3.5. Magnetic Analysis 

**Figure 8:**
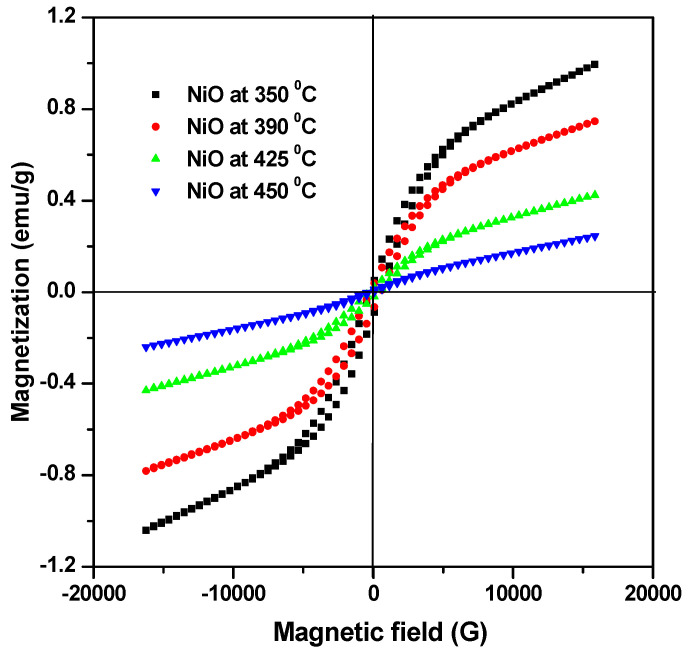
M-H loops for NiO NFs annealed at different annealing temperatures.

The magnetic performance as a function of heat treatment of precursor NiO NPs was measured by vibrating sample magnetometer analysis (VSM). Figure 8 shows that the study investigates how the magnetic properties change with different heat treatments of precursor NiO nanoparticles and how the annealing at different temperatures (350 °C, 390 °C, 425 °C, and 450 °C) affects the magnetic behavior of NiO nanoparticles. Hysteresis loops are observed in M-H curves of annealed (350 °C and 390 °C) precursor NiO NPs, indicating the presence of ferromagnetic Ni domains. However, NiO nanoparticles annealed at higher temperatures (425 °C and 450 °C) show a linear M-H curve, suggesting paramagnetic behavior. This shift from ferromagnetic to paramagnetic behavior of NiO nanoparticles can be interpreted as follows: at lower temperatures, NiO nanoparticles may exhibit some degree of ferromagnetic ordering due to uncompensated spins at the surface or defects. As the annealing temperature increases, these defects may anneal out, reducing the magnetic interactions and leading to a transition from ferromagnetic to paramagnetic behavior. Magnetic properties such as ferromagnetism or paramagnetism are closely tied to structural properties such as shape, size, and crystallinity of the nanoparticles. Bulk NiO is antiferromagnetic, but as particle size reduces from bulk to nano, different magnetic behaviors (like weak ferromagnetism, superparamagnetism, and spin glass behavior) can emerge due to spin disorder caused by broken exchange bonds and Ni vacancies [3,35,36,37]. The crystallite size of NiO nanoparticles increases with higher heat treatment temperatures. Increased crystallite size is associated with a reduced surface-to-volume ratio, which favors paramagnetic behavior in NiO nanoparticles [5,6].

### 3.6. Photocatalytic Performance

**Figure 9:**
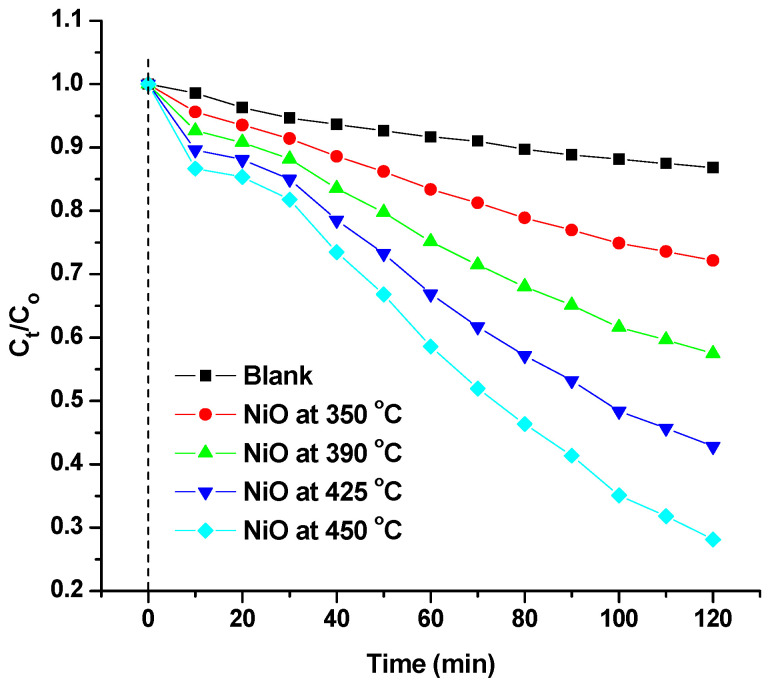
Photocatalytic degradation rates of MB with NiO NPs at different annealed temperature.

After the dark adsorption procedure, the MB photodegradation tests of the NiO sequences were evaluated under simulated sunshine irradiation. Figure 9 displays the outcomes of two hours of elimination efficiency adsorption as the precursor of NiO that was annealed at 350, 390, 425, and 450 °C. The NiO series exhibits increased photocatalytic activity with higher annealing temperatures, but MB is stable under irradiation without any photocatalyst, according to this figure. Surprisingly, the photocatalytic performance of MB increased as the annealed temperatures rose, with NiO nanoparticles exhibiting the best performance at 450 °C. At 450 °C, irradiation caused NiO nanoparticles to degrade with a 72.4% efficiency. Higher charge separation efficiency, stronger photo adsorption, and better adsorption performance of MB molecules may be responsible for the increased efficiency.

**Figure 10:**
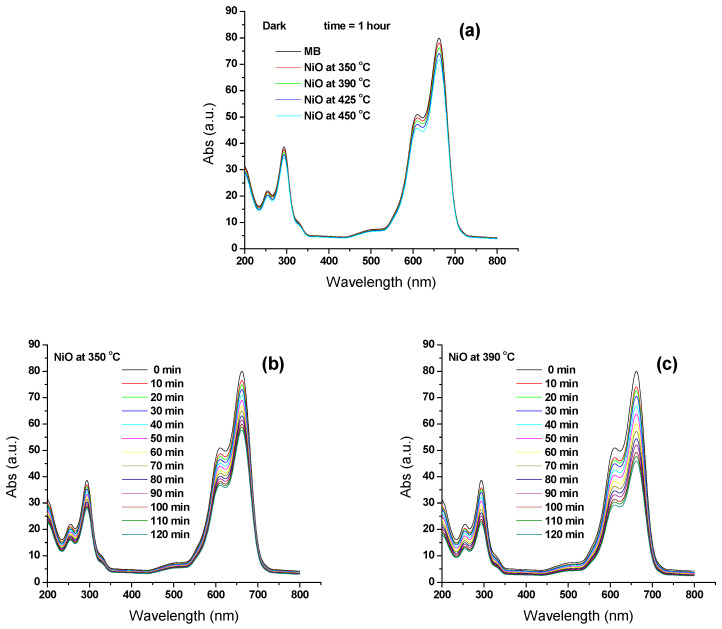

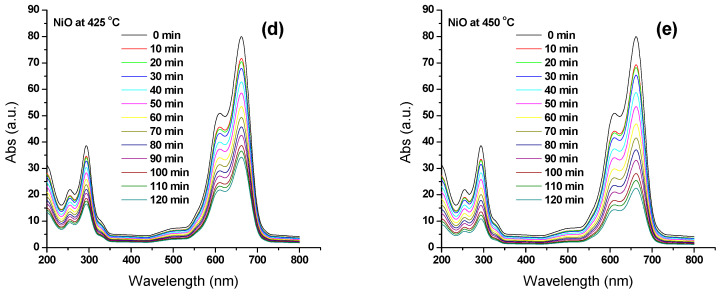
Photocatalyst performance of the synthesized samples under (**a**) dark light for NiO samples at different annealed temperature for 1 h. And visible light irradiation time (**b**) NiO at 350 °C (**c**) NiO at 390 °C, (**d**) NiO at 425 °C and (**e**) NiO at 450 °C. The black spectrum was measured from the MB solution used for the experiments.

Before photocatalytic testing, all samples in Figure 10a show a very slight change in darkness. Prior to photocatalytic testing, the pure MB solution was stabilized by exposure to white light. The solutions’ UV-Vis spectra did not show any notable modifications. The NiO annealed (at 350, 390, 425, and 450 °C) samples had photocatalytic activity in MB degradation, as shown in Figure 10b–e. However, when MB and NiO suspensions were exposed to visible light irradiation, a significant quantity of MB was destroyed as a result of the production of highly reactive OH radicals, which serve as potent oxidants [7,38,39,40]. At annealing temperatures of 350 °C, 390 °C, and 425 °C, respectively, the MB degradation was approximately 28.4%, 43.6%, and 57.5% for the catalyst. The sample that was annealed at 450 °C, however, exhibits the maximum photocatalytic activity, reaching up to 72.4% after being exposed to visible light. As a result, it is seen that when the catalyst’s annealing temperature rises, the rate of MB degradation also increases. As seen in Figure 7, the band gap of NPs decreases as the heat treatment of NiO NPs rises. The easier charge transfer from the valence band to the conduction band caused by the reduction favors better electrical contact between the NiO and MB molecules. The smaller band gap makes it simpler to transfer charge from the catalyst to the MB molecules’ Fermi level.

This improves the dye degradation efficiency by causing a number of MB molecules to adsorb to the catalyst’s surface [41]. The band gap was typically considered to be the primary factor in explaining the photocatalytic activity of semiconductors. We may anticipate that NiO NPs produced at a low annealing temperature will only react to UV light because of the wide band gap of NiO. However, in this instance, the oxygen species in the catalyst can be used to explain the visible light absorption. In order to cause absorption in the visible area, temperature produces an intermediate energy band adjacent to the valence band. An electron is stimulated from the valence band to the conduction band when the NiO NPs in the catalytic system are exposed to visible light through the energy absorbed. Positively charged holes in the valence band make good oxidants, whereas negatively charged electrons in the conduction band make good reductants.

NiO catalytic behavior is the same as TiO_2_ and ZnO in the dark, but NiO’s activity under visible light may be superior if optimally annealed. In general, however, TiO_2_ and ZnO are more commonly used due to their well-established photocatalytic properties [42,43,44]. 

### 3.7. Mechanism for the MB Degradation

When NiO is exposed to visible light, photons are absorbed by the material. This energy excites electrons from the valence band to the conduction band, creating electron-hole pairs. The excitation of NiO generates free electrons (e^−^) in the conduction band and holes (h^+^) in the valence band. The generated electrons and holes can interact with the surrounding environment to produce reactive oxygen species (ROS) such as hydroxyl radicals (OH), superoxide anions (O_2_^−^), and hydrogen peroxide (H_2_O_2_). The holes (h^+^) in the valence band are highly oxidative and can react with water molecules or hydroxide ions present in the solution to produce hydroxyl radicals:

h^+^ + H_2_O → OH + H^+^

h^+^ + OH^−^ → OH

The generated hydroxyl radicals (OH) are extremely reactive and can interact with the MB dye molecules. The MB dye has several functional groups, including the methylene blue chromophore, which are susceptible to oxidative degradation. Hydroxyl radicals oxidize the MB dye by breaking the dye’s chemical bonds. This leads to the decolorization of the dye as the aromatic structure and chromophore of MB are disrupted. During the degradation process, various intermediate products may form. These are typically smaller organic compounds or fragments that can further degrade or eventually mineralize into simpler molecules. Ideally, the degradation process should lead to complete mineralization of MB into carbon dioxide (CO^2^), water (H^2^O), and inorganic ions. The surface of NiO also plays a role in the photocatalytic process. The MB dye may adsorb onto the NiO surface, bringing it closer to the reactive sites where the hydroxyl radicals can effectively interact with it. The efficiency of the NiO photocatalyst depends on its surface properties, which can be influenced by factors such as annealing temperature. Well-annealed NiO with improved surface area and active sites can enhance the degradation efficiency.

In summary, the superior photocatalytic performance of paramagnetic NiO nanoparticles compared to ferromagnetic NiO nanoparticles can be attributed to their higher surface area, favorable electronic structure for photocatalysis, improved charge carrier dynamics, and overall better stability under photocatalytic conditions. These factors collectively contribute to their effectiveness in applications such as environmental remediation and solar energy conversion.

Finally, the study indicates that higher annealing temperatures improve the photocatalytic properties of NiO. Elevated annealing temperatures likely enhance the crystallinity and surface characteristics of NiO, thereby increasing its ability to generate reactive OH radicals and, consequently, its photocatalytic efficiency. Future work will assess the stability of NiO nanoparticles (NPs) by analyzing XRD patterns and TEM images to determine if the NPs degrade or alter upon interacting with MB dye molecules. This will involve comparing XRD patterns before and after interaction with MB, looking for shifts or changes in peak positions and intensities that could signify structural transformations or degradation. Additionally, TEM images will be examined to detect any variations in particle size, shape, or aggregation, providing insights into the material’s physical stability.

## 4. Conclusions

We have proposed an experimental method for producing NiO nanoparticles that relies on the thermal disintegration of the precursor nickel hydroxide nanoparticles and the chemical reduction of nickel chloride with hydrazine at room temperature. TGA analyses were performed in an environment between 25 and 550 °C. It is clear from the TGA curve that there was more than one stage of weight loss. Above 435 °C, obviously, the weight loss becomes fairly slight, indicating that the decomposition is complete and the formation of NiO nanoparticles is nearly complete.

The NiO NPs are crystalline, according to the XRD data, and the diffraction peaks can be accurately categorized as having a face centered cubic (FCC) structure. The treated materials’ predicted crystallization size rose from 6 to 18 nm. TEM was used to determine the nanoparticles’ size and shape. The average crystallite size computed from the XRD pattern and the mean particle size measured by TEM values are reasonably in accord. With rising annealing temperatures, it is shown that the absorption maximum, λ_max_, exhibits red shifts to longer wavelengths from 327 nm to 409 nm.

The magnetic behavior as a function of heat treatment of precursor NiO NPs was measured by vibrating sample magnetometer analysis (VSM). The increase in heat treatment causes a change in the magnetic behavior of NiO nanoparticles from weak ferromagnetism (which indicates some degree of magnetic ordering) towards paramagnetism (where magnetic moments are randomly oriented). In a similar vein, NiO NPs have the highest photocatalytic activity for the degradation of MB in the presence of visible light at a higher annealing temperature. The decreased band gap of the catalyst was blamed for the increase in MB’s degradation efficiency. As a result, NiO NPs can be employed as a powerful photocatalyst for the removal of MB from wastewater. It is known that NiO NPs’ annealing temperature significantly affects adsorption and photocatalysis by regulating particle and grain sizes. These NiO NPs can also be employed in wastewater treatment methods.

## Data Availability

The raw data supporting the conclusions of this article will be made available by the authors on request.
